# Hepatitis A virus induced acute acalculous cholecystitis diagnosed postoperatively: Case report

**DOI:** 10.1016/j.ijscr.2024.109687

**Published:** 2024-04-23

**Authors:** Omar Tabbikha, Mahmoud Dasuki, Anthony Kanaan, Bader Ali, Ribal Aby Hadeer, Raja Wakim

**Affiliations:** Department of General Surgery, Mount Lebanon Hospital University Medical Center, Faculty of Medicine and Medical Sciences at University of Balamand, Beirut, Lebanon

**Keywords:** Hepatitis A virus, Acalculous cholecystitis, Laparoscopic cholecystectomy, Case report

## Abstract

**Introduction:**

acute acalculous cholecystitis (AAC) is defined as gallbladder inflammation without the presence of stones. Contrary, hepatitis A virus (HAV) can present with different symptoms; however, HAV causing and presenting as AAC is rare.

**Case presentation:**

41-year-old previously healthy patient presented with right upper quadrant abdominal pain. The pain was persistent and associated with vomiting and laboratory tests showed elevated bilirubin. Laparoscopic cholecystectomy showed inflamed gallbladder with no stones and intraoperative cholangiography showed no abnormalities. Day one post-operation, while the pain resolved, labs showed elevated liver function tests and hepatitis workup showed acute HAV infection attributing her presentation to HAV induced AAC.

**Discussion:**

AAC is usually caused by stasis of the gallbladder due to different causes; however, HAV induced AAC has been rarely reported. While cholecystectomy is the mainstay treatment for AAC, this might not be the case for HAV induced AAC. For instance, unless there is necrotic gallbladder or persistence of symptoms, AAC can be managed conservatively in this case. Even though our diagnosis was cleared post-operatively, had we knew the diagnosis of HAV induced AAC before, we would have still opt for surgery due to the severity and persistence of pain.

**Conclusion:**

More cases should be reported and more studies should be done to further define the presentation and management of HAV induced AAC.

## Highlights

• Hepatitis A virus as acute acalculous cholecytitis cause has been rarely reported.
• Cholecystectomy might not be the treatment when cholecytitis is caused by hepatitis A virus.
• Hepatitis A induced acute acalculous cholecystitis can be managed conservatively.
• More cases should be reportedto further define the management of hepatitis A induced acute acalculous cholecystitis.

## 1 Introduction

Acute acalculous cholecystitis (AAC) is defined as an acute inflammatory disease of the gallbladder without the evidence of gallstones [1]. It accounts only for 5–10 % of all causes of acute cholecystitis and it often presents postoperatively in trauma centers and intensive care units (ICU) [1,2]. On the other side, hepatitis A is generally an acute, self-limited infection of the liver caused by an enterically transmitted hepatitis A virus (HAV) [3]. While most cases of HAV are asymptomatic, some present with diarrhea, abdominal pain, fever, anorexia, nausea, and vomiting [4]. Nevertheless, HAV causing and presenting as symptomatic AAC is very rare and only 29 reports have linked them together [5,6]. Here, we present a case of HAV induced AAC which presented to our university hospital and was diagnosed postoperatively. To note, our case is reported in line with the SCARE criteria [7].

## 2 Case presentation

**Fig. 1:**
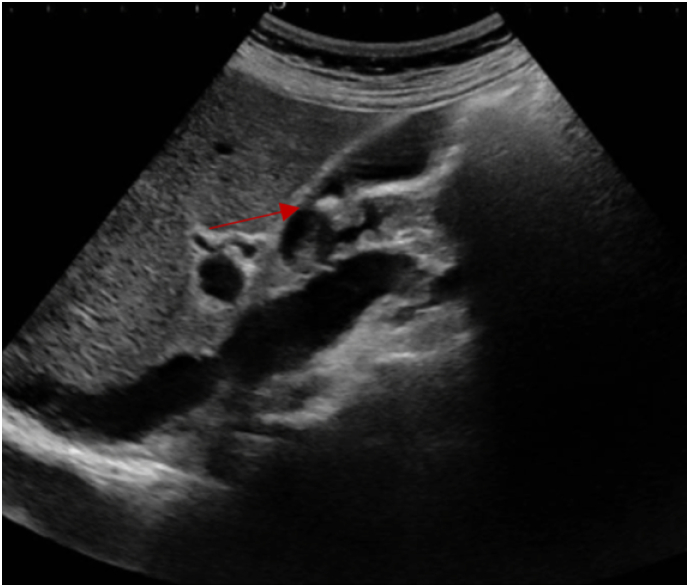
Gallbladder ultrasound done at The Peripheral Hospital. Red arrow pointing to the fold that was initially considered a stone at the peripheral hospital. (For interpretation of the references to colour in this figure legend, the reader is referred to the web version of this article.)

**Fig. 2:**
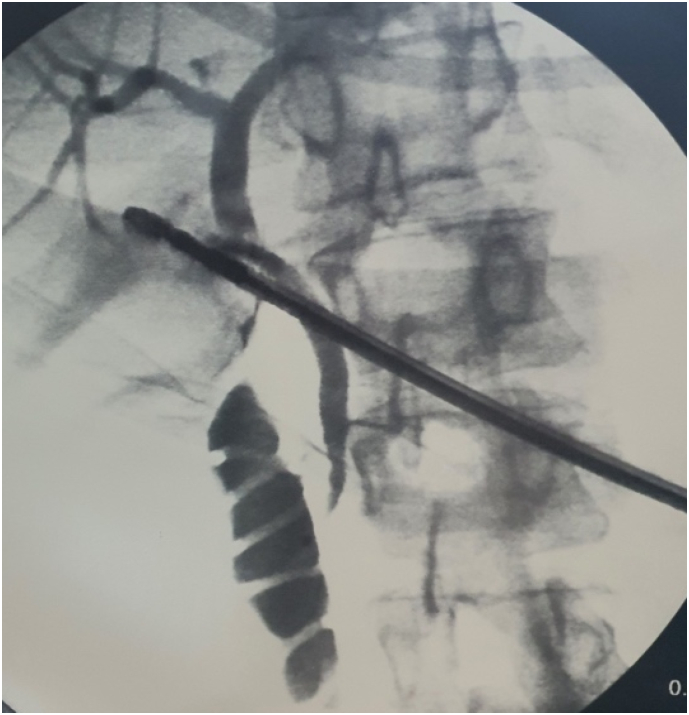
Intraoperative cholangiography showing no filling defects.

41-year-old previously healthy caucasian female patient presented to our emergency department (ED) for severe right upper quadrant (RUQ) abdominal pain that had exacerbated six hours prior to presentation. The pain had started two days prior to presentation for which she visited the ED in a peripheral hospital. There, blood analysis (Table 1) was done and showed neutrophilic shift, elevated C-reactive protein (CRP), and slightly high levels of the transaminases. Abdominal ultrasound (US) (Fig. 1) was done and showed a mildly distended gallbladder with a wall thickening of 4 mm and the presence of a 10 mm stone. There were no pericholecystic fluid nor dilation of the bile ducts. Sonographic Murphy's sign was negative. The patient was considered to have biliary colic and was offered to be admitted for further evaluation and to undergo laparoscopic cholecystectomy; however, her symptoms decreased in intensity on intravenous analgesics meanwhile, for this reason she refused to be admitted and got discharged. Nevertheless, the next day the pain returned and became severe in intensity, constant in nature, not radiating, post-prandial, and not alleviating by over the counter pain medications for which she presented to our ED. This time, the pain was also associated with four episodes of vomiting which developed over the past two days, and she did report one week history of myalgia, nausea, anorexia, and generalized fatigue. The patient reported no fever or chills and normally consistent and colored bowel movements, but did notice recent urine darkening. To note, the patient takes no medications, has no pertinent past medical family history, is not a smoker, and drinks alcohol occasionally. Moreover, she underwent hysteroscopic myomectomy for uterine fibroids two weeks ago; in addition, open appendectomy, adenoidectomy, and tympanoplasty were done at the age of 14 year old, 15 year old, and 22 year old, respectively. On physical examination, vital signs were normal with a temperature of 37.3 °C, blood pressure of 105/68 mmHg, and heart rate of 73 beat per minute. She was pale but not icteric, and the abdominal examination revealed soft abdomen but with RUQ and epigastric tenderness and with a positive Murphy's sign. Blood test showed normal complete blood count, electrolytes, and lipase with only elevated CRP but lower than the previous value; however, this time, total and direct bilirubin blood tests were also ordered that showed elevated values (Table 1). Therefore, taking into consideration the patient's persistent severe RUQ pain with positive Murphy's sign, the presence of a 10 mm gallbladder stone in the US done two days ago, and the elevated level of CRP and bilirubin, plan was made to undergo laparoscopic cholecystectomy with intraoperative cholangiography (IOC) and possible bile duct exploration or intraoperative endoscopic retrograde cholangiopancreatography (ERCP) if needed for acute calculus cholecystitis with possible stone migration. Intraoperatively, an inflamed gallbladder was identified, and the smoothly done cholangiogram (Fig. 2) showed no abnormalities or filling defects in the biliary tree (Fig. 2). Furthermore, the cystic duct and artery were clipped and cut and the gallbladder was dissected carefully from the liver bed with no injury; however, when removed and opened, no stones nor sludge were found in the gallbladder. At this stage the etiology of AAC was highly suspected rather than a calculus cholecystitis with a migrated stone. The last is mainly due to the presence of gallbladder inflammation without an obstructing stone and the difficulty of a 10 mm gallbladder stone or any stone large enough to cause cholecystitis to migrate through the common bile duct (CBD) either spontaneously without being stuck or causing bile duct dilatation or during cholangiography without extensive CBD flushing; moreover, when the US done in the peripheral hospital was reassessed at our institution retrospectively, it was found that the suspected reported stone could had been mistaken by a folded gallbladder wall especially due to the absence of a clear posterior shadowing. Post operation day one, while the patient's abdominal pain was already resolved, laboratory tests (Table 1) were ordered including full liver function test for further assessment and monitoring. Laboratory tests showed elevated liver function tests; therefore, taking into consideration this elevation, the presence of acalculous cholecystitis with no clear etiology, and the one week history of fatigue and myalgia, the presence of viral hepatitis was highly suspected. Therefore, complete viral hepatitis workup was ordered that all turned to be negative except for anti-HAV immunoglobulin M which was found to be 8.82 S/Co indicating acute viral hepatitis A. Consequently, the etiology of her previous symptoms were attributed to HAV induced AAC. The patient was maintained on supportive therapy: intravenous hydration, anti-emetics, and low-fat diet. On the second day of hospitalization, the patient's condition was significantly improved and she was discharged home on supportive therapy. To note, the histopathology showed gallbladder with cholesterolosis that is edematous, congested, and with polymorphous leukocytic infiltrates showing signs of cholecystitis and no malignancy.

**Table 1:** Patient's laboratory values. WBC: white bloot cells, New: neutrophils, lym: lymphocytes, Hb: hemoglobin, CRP: C-reactive protein, Cr: creatinine, AST: aspartate transaminase, ALT: alanine transaminase, bili T/D: bilirubin total/ direct, ED: emergency department, POD: post operation day.

	WBC x10^3^/uL	Neu %	Lym %	Hb g/dL	Platelets x10^3^/uL	CRP mg/L	Cr mg/dL	AST IU/L	ALT IU/L	Bili T/D mg/dL
Peripheral ED	3.6	77.1	14.4	12.7	207	25	0.75	111	96	X
On presentation	8.2	47	27	13.8	347	8	0.56	X	X	3.81/2.9
POD 1	11.7	48	28	12.3	338	X	0.58	790	1292	4.45/3.3

## 3 Discussion

Acute inflammation of the gallbladder without the evidence of cholelithiasis is known as AAC [8]. Its presentation can vary from asymptomatic, to typical RUQ abdominal pain with positive Murphy's signs, reaching frequently gangrene, perforation, and empyema; therefore, it can be associated with high morbidity and mortality [9]. In our case, the patient presented with signs of acute cholecystitis (constant RUQ pain and positive Murphy's sign), but the diagnosis of AAC was not made until postoperatively, when an inflamed gallbladder was noted with no stones and normal IOC. AAC is usually caused by stasis of the gallbladder, which results in inflammation and ischemia of its wall due to the build-up of intraluminal pressure [10]. Stasis can be due to parenteral nutrition, analgesics, or certain drugs such as thiazides, ceftriaxone, erythromycin, or ampicillin. Moreover, other causes include bacterial infection (either retrograde or from blood), severe dehydration, and accumulation of certain viral metabolites; for instance, AAC is a very rare complication of HAV, and can occur at any period of the current viral infection [11,12]. In our case, the patient had none of the above mentioned etiologies for AAC except for hepatitis A which was diagnosed directly postoperatively; hence, our patient's pain was attributed to AAC due to HAV.

HAV has been extensively studied throughout history. It is transmitted by fecal oral-route and mostly causes a self-limited disease, if no complications arise. HAV is one of several viral etiologies causing hepatitis. Most have overlapping symptoms and are hard to differentiate from each other without serologic examinations. Symptoms include fever, abdominal pain, jaundice, dark urine, myalgia, nausea, and vomiting [13]. In acute setting, all viral hepatitis have shown marked elevation in liver function tests (LFT), total and especially direct bilirubin, and alkaline phosphatase. Transaminases have typically been described as >500 IU/L, even reaching numbers as high as 5000 IU/L, and have been noted to be elevated before any bilirubin rise is detected. Moreover, ALT has showed to be at higher levels than AST [14]. In our case, our patient did report one week history of dark urine, myalgia, nausea, vomiting, fatigue and RUQ abdominal pain; however, since she had positive Murphy's sign, and a previously reported 10 mm gallbladder stone, acute calculus cholecystitis was initially our diagnosis and hepatitis A was not tested until AAC was found and HAV as its cause was suspected due to the patient's symptoms and postoperative labs; for instance, as described above, our patient did have disturbed LFTs with ALT > AST and elevated both direct and total bilirubin (Table 1).

While cholecystectomy is the mainstay management for AAC in general and cholycystostomy is the lifesaving alternative for unstable patients [15], this might not always be the case in HAV induced AAC. The discovery of HAV particles invading the gallbladder epithelial lining with multiple cell-mediated immunogenic cells suggests a distinct pathogenesis specific to hepatitis A viremia. This is further established as HAV induced AAC symptoms dissipate after treating viremia with only symptomatic management in most published cases. Furthermore, a significant reduction in gallbladder wall thickness and diameter is seen reaching normal levels with only conservative management. Thus, treatment mainstay is only conservative management without any surgical interventions; unless necrotic gallbladder is suspected, patient fails to improve, or becomes unstable. Patients with AAC caused by HAV have been mainly presenting as mild to moderate cases in outpatient setting leading to higher confidence among physicians in treating them conservatively, as opposed to severely ill ICU patients diagnosed with AAC that seem to lean physicians towards more invasive approaches [16]. In our case, the patient presented to the ED two times for severe abdominal pain which became non resolving on over the counter pain medications; therefore, even though calculus cholecystitis was our initial diagnosis and HAV induced AAC diagnoses was only made postoperatively, laparoscopic cholecystectomy would have also been our plan if the HAV induced AAC diagnosis was made preoperatively due to the failure of improvement and the severity of pain.

## 4 Conclusion

Even though AAC is a common well studied disorder, HAV as its cause is very rare and not well described in literature. Due to ours and others few reported cases of HAV as a cause of AAC, one has to have high index of suspicion for it especially in patients with no obvious cause of AAC. Nevertheless, had we had the last diagnosis prior to the operation, we would have been in a dilemma of whether to operate for failure of improvement, which would have been our choice, or keep only supportive care since HAV induced AAC can be managed conservatively. Therefore, we recommend more cases to be reported and more studies to be done to further define the presentation and management of HAV induced AAC and indicate accurately the indications for its surgical management.

## Consent

Written informed consent was obtained from the patient for publication of this case report and accompanying images. A copy of the written consent is available for review by the Editor-in-Chief of this journal on request.

## Informed consent

An informed consent was signed by the patient in order to authorise access on her medical records and for the completion of this work.

## Ethical approval

Case report approved for publishing by ethical committee at mount Lebanon hospital, University Medical Center, and Head of General Surgery division on November 1, 2023.

## Funding

Faculty of Medicine and Medical Sciences, University of Balamand.

## Author contribution

Omar Tabbikha (first author and corresponding author), Mahmoud Dasuki, Anthony Kanaan, Bader Ali, Ribal Aby Hadeer, Raja Wakim.

## Guarantor

Raja Wakim.

## Research registration number

1.Name of the registry: Clinicaltrials.gov.

2.Unique identifying number or registration ID: NCT06191471.

3.Hyperlink to your specific registration (must be publicly accessible and will be checked): https://clinicaltrials.gov/study/NCT06191471?cond=NCT06191471&rank=1.

## Conflict of interest statement

The authors report no conflict of interest.
